# Loss of Radial Height in Patients Undergoing Fixation with a Distal Volar Anatomical Locking Plate: A Retrospective Study in a Public Institution

**DOI:** 10.1055/s-0045-1814404

**Published:** 2025-12-30

**Authors:** Heitor Teixeira Alves Carvalho, Yves Pacheco Dias March e Souza, Felipe Pinheiro da Silva, Érica Maciel Heringer, João Carlos Ostermeir Silva Pereira, Saulo Fontes de Almeida

**Affiliations:** 1 Medical Residency Program, Instituto Nacional de Traumatologia e Ortopedia, Rio de Janeiro, RJ, Brazil; 2 Hand Surgery Center, Instituto Nacional de Traumatologia e Ortopedia, Rio de Janeiro, RJ, Brazil

**Keywords:** bone plates, fracture fixation, internal, radius fracture, distal, fixação interna de fraturas, fraturas distais do rádio, placas ósseas

## Abstract

**Objective:**

To evaluate and quantify the frequency and magnitude of late loss of radiographic parameters following volar locking plate fixation of distal radius fractures.

**Methods:**

The present is a retrospective, cross-sectional study that radiographically analyzed 65 wrists operated on between 2020 and 2024 at a public institution, evaluating postoperative loss of radial height.

**Results:**

The results showed a median loss of 0.60 mm, with 13.8% of patients experiencing losses greater than 2 mm, and 75.4% experiencing lower losses. No evidence was found that sex statistically significantly affects this incidence after the Wald test.

**Conclusion:**

Despite the effectiveness of the volar locking plate in maintaining reduction, loss of radial height still occurs, especially in older patients with more complex fractures. The present study corroborates the use of this osteosynthesis technique, a fundamental tool in the management of complex fractures of the distal third of the radius, to restore and maintain the anatomy of the region.

## Resumo

**Objetivo:**

Avaliar e quantificar a frequência e magnitude da perda tardia de parâmetros radiográficos de redução de fraturas de rádio distal em pacientes operados com placa volar bloqueada.

**Métodos:**

Este é um estudo retrospectivo e transversal que analisou radiograficamente 65 punhos operados entre 2020 e 2024 em uma instituição pública, avaliando a perda de altura radial no seguimento pós-operatório.

**Resultados:**

Os resultados mostraram uma perda mediana de 0,60 mm, com 13,8% dos pacientes apresentando perdas superiores a 2 mm e 75,4% perdas inferiores. Não encontramos indícios de que o sexo do paciente afete de forma estatisticamente significativa essa incidência após o teste de Wald

**Conclusão:**

Apesar da eficácia da placa volar bloqueada na manutenção da redução, a perda de altura radial ainda ocorre, especialmente em pacientes mais idosos e com fraturas mais complexas. O presente estudo corrobora a utilização dessa técnica de osteossíntese que mostra-se uma ferramenta fundamental na abordagem das complexas fraturas do terço distal do rádio, a fim de restaurar e manter a anatomia da região.

## Introduction

Distal radius fractures are the most frequent among those of the upper limbs, corresponding to 17% of all skeletal fractures and 75% of forearm fractures. 1 They have a bimodal distribution, in young people through high-energy trauma, and in the older population with low-energy trauma. 2 In recent years, an increase in distal radius fractures has been observed, mainly as a result of population aging, with them being the second most common type of fracture in this age group. 3

Specific fracture analysis and treatment planning require knowledge of the region's normal anatomy. 4 The distal end of the radius is part of the wrist joint along with the distal end of the ulna and carpal bones, with an anatomical and biomechanical balance between the bone, muscle tendon, and ligament structures that allow for hand functionality. 5

The distal end of the radius has a complex anatomy, with concavities and angulations in different planes. It has three concave articular facets: the scaphoid and lunate fossae and sigmoid notch, where it articulates with the scaphoid, lunate, and head of the ulna, respectively. The lunate fossa is critical to the stability of the radiocarpal and radioulnar joints and involves bone and ligament relationships. 5 The dorsal aspect of the distal end of the radius features the Lister tubercle, a bony prominence that can range from 4 to 10 mm and acts as a fulcrum for the extensor pollicis longus tendon. 6

**Fig. 1:**
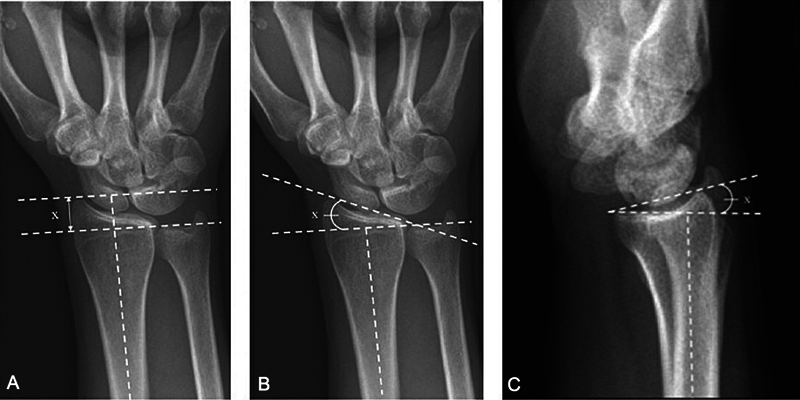
( **A** ) Mean radial height (X) is 11 to 12 mm. ( **B** ) Mean radial inclination (X) is 22 to 23 ^o^ . ( **C** ) The volar tilt (X) is 11 to 22 ^o^ . (From Patel SP, Rozental TR. Distal radius fractures: biomechanics and classification. Hand Surgery Update VI. © 2016 American Society for Surgery of the Hand).

The distal end of the radius has inclinations and measurements that are evaluated on radiographs in neutral posteroanterior (PA) (radial inclination, radial height, and ulnar variance) and profile (volar inclination or tilt) incidences ( Fig. 1 ).

The radial inclination is the angle between a line tangent to the articular surface of the radius, from the top of the radial styloid to the ulnar corner of the radius, and another line perpendicular to the longitudinal axis of the radius shaft, with a mean of 23 degrees. 4

The radial height, used in the evaluation of radius shortening after fracture, can be obtained by a radiograph in PA incidence. Two lines are drawn perpendicular to the long axis of the radius, one at the tip of the radial styloid and the second at the ulnar corner of the distal radial articular surface; their normal value is about 11 to 12 mm. 7

The ulnar variance is evaluated by a line tangent to the subchondral surface of the radiolunate joint and another tangent to the most distal portion of the articular surface of the ulna, with a mean of 1 mm. 8

The radial volar tilt is the angle formed between a line that touches the volar and dorsal lips of the lunate fossa and another line perpendicular to the longitudinal axis of the radius, with a mean of 11 degrees. 4

Evaluating these parameters radiologically has proven to be a reliable and reproducible method. 9

These radiographic parameters are fundamental to evaluate the correct anatomical and functional restoration of the distal third of the radius after fractures and their respective surgical reconstructions. 10

Distal radius fractures present with a wide range of patterns, and when surgical treatment is required, although several techniques have already been described, open reduction and internal fixation with a locking volar plate has become the method of choice. 11 The locking volar plate technique enables placement of screws in a subchondral position, providing joint surface support and reducing loss of reduction in fractures with dorsal comminution. 12

The application of locking plates on the volar surface of the radius allowed stable fixation of intra- and extra-articular fractures, enabling early wrist mobilization and faster joint functional recovery, with superior results compared to non-locking plates, both volar and dorsal. 13 Despite this, they have complication rates ranging from 3 to 36%, according to the literature, 14 with loss of reduction being the most significant among the possible complications. 15

There is a consensus among surgeons that the best postoperative results are obtained by reestablishing preoperative radiographic anatomical parameters. Loss of postoperative reduction, especially of radial height, is a complication that can lead to pain, instability, and loss of strength. 16

The present study aims primarily to evaluate and quantify the frequency and magnitude of late loss of radiographic parameters following volar locking plate fixation of distal radius fractures. Secondly, we aim to correlate the radiographic parameters with the patients' chronology and epidemiological data.

## Materials and Methods

The present study is a retrospective and cross-sectional analysis, approved by the Research Ethics Committee CAAE: 82364524.2.0000.5273, of patients with distal radius fractures who underwent osteosynthesis in our service between 2020 and 2024. The anatomical volar locking distal radius plate APTUS wort 2.5 (Medartis) was used as an implant.

The inclusion criteria were patients undergoing distal radius surgery with APTUS wort 2.5, minimum follow-up of 3 months, and patients over the age of 18 years.

The exclusion criteria were patients with associated fractures in the same limb, use of synthetic material in addition to the locking plate, and patients without a postoperative radiographic record available in the digital imaging system.

Patients with a time interval of up to four weeks between fracture and surgery were evaluated in the present study.

Through the digital filter “FRACTURE OF THE DISTAL END OF THE RADIUS/ FRACTURE OF THE FOREARM”, the wrists operated in our service were selected during the established period. After radiographic and medical record evaluation, 114 wrists were selected for the study.

The radiographs used are standardized by the institution. In the current study, we used the true AP incidence, 1 m away from the book, 44 kilovolts (KV), and 3.2 milliamperes per second (mA/s).

**Fig. 2:**
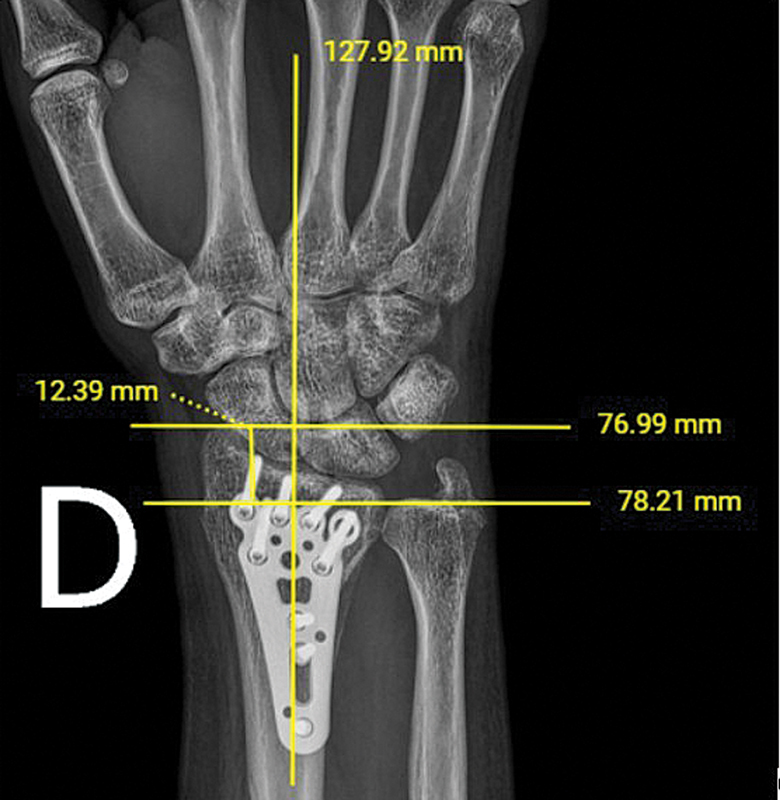
Radial height in the immediate postoperative period.

Radial height was measured on the first postoperative radiograph and on subsequent postoperative radiographs using the institution's digital imaging system ( Figs. 2 3 ). Data such as sex, age, fracture laterality, and date of surgery were also collected. The data obtained were input into a spreadsheet and the measurements were performed by four evaluators.

**Fig. 3:**
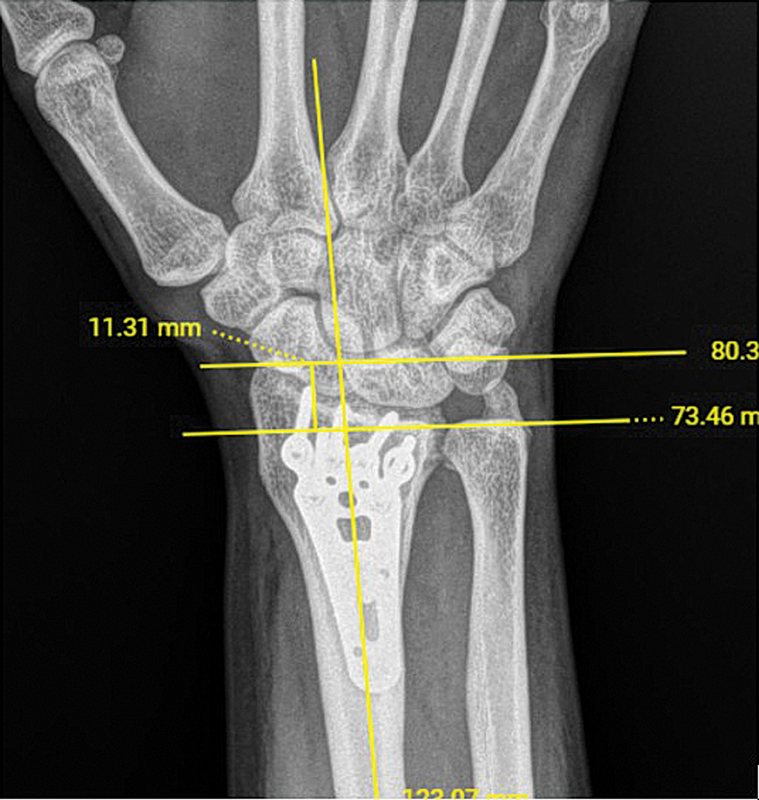
Radial height change over a period of six months.

Postoperative patients with distal radius fractures return at 3 weeks, 3 months, 6 months, and 1 year as a follow-up protocol. For the present study, we recruited patients with at least 2 radiographs within the past 3 months.

To standardize the evaluation, the incongruity, displacement, energy, age, and lesions (IDEAL) classification system was used, which analyzes five variables. For each one of them, a score of 0 to 1 is assigned based on the presence or absence of the factors, totaling 5 points, with type I being from 0 to 1 point; type II from 2 to 3 points, and type III from 4 to 5 points.

To score 0 or 1, the parameters according to Table 1 were used.

**Table 1:** IDEAL classification

	Characteristics	0 points	1 point
**I**	Age	< 60 years	> 60 years
**D**	Deviation	No	Deviation that needs reduction
**E**	Energy 1	Low	High
**A**	Articular incongruity	No	Incongruity or gap > 2 mm
**L**	Associated injuries 2	Absent	Present

**Notes** : 1. LOW: fall from own high/HIGH: others.

2. Open fractures/carpal bone fractures/carpal instability/distal ulna fracture.

**Table:** 

Classification	Score	Description	Treatment	Prognosis
I	0–1 points	Stable	Conservative	Good
II	2–3 points	Potentially unstable	External fixation, percutaneous K-wire fixation, and plate fixation	Fair
III	4–5 points	Complex	Associated methods, bone grafting	Poor

## Statistical analysis

The variation in radial height relative to the first postoperative radiograph, expressed in mm, was calculated. Categorical data are presented as absolute occurrence (percentage), while numerical data, which did not present normal distribution according to the Shapiro-Wilk test (alpha of 0.05), are presented as median (interquartile range [IQR]). The incidence of loss in radial height greater than 2 mm was calculated, and the effect of sex on this incidence was quantified by the odds ratio (OR), obtained in conventional logistic regression, and evaluated by the Wald test. The analyses and figures were developed using routines written in Python 3.10 (Python Software Foundation), with a significance level of 0.05.

## Results

Data from 114 wrists were analyzed; 65 with a minimum follow-up of 3 months were included. Data were obtained from 62 patients, including 3 cases of bilateral fracture.

**Fig. 4:**
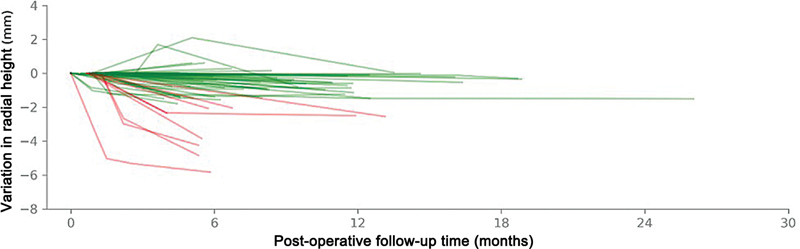
Variation in radial height of the wrists followed-up. Each wrist is represented by an individual line, with those with height loss greater than 2 mm shown in red and the others in green.

Among unilateral cases, the left side was affected in 41 cases and the right in 18. The median age was 54 years, with IQR of 46 to 61; 33 (53.2%) were women, and 29 (46.8%) were men. The median follow-up time was 6 months (IQR 4-10), ranging from 3 to 18 months ( Fig. 4 ).

**Fig. 5:**
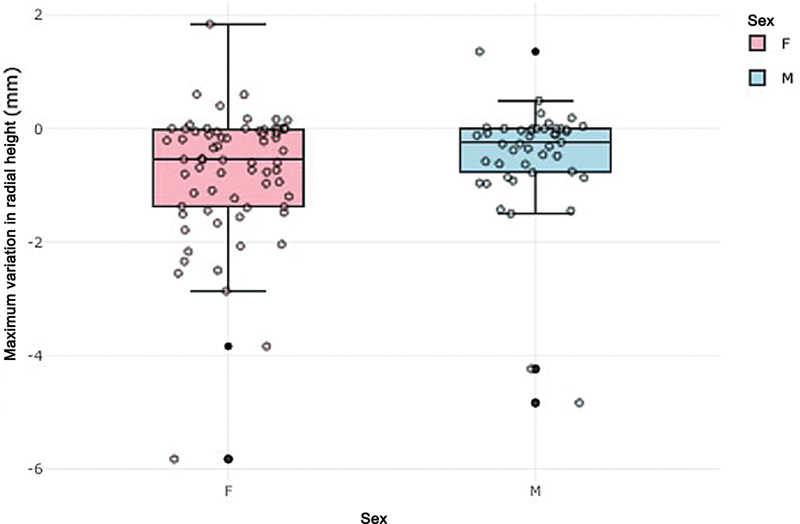
Postoperative variation of radial height (in mm), by sex.

Considering the last measurement obtained for each wrist, a height loss of 0.60 mm (0.14–1.43) was observed, with a maximum of 5.83 mm ( Fig. 1 ). No level of depression was detected in 7 (10.8%) wrists, while 49 (75.4%) presented detectable losses of up to 2 mm, and 9 (13.8%) presented greater losses during the period analyzed ( Table 2 ). Two cases of loss greater than 2 mm were observed among men and 7 among women, corresponding to 6.7% and 20.0%, respectively. No statistically significant effect of sex on this incidence was found using the Wald test (OR = 3.5; *p* = 0.138) ( Fig. 5 ).

**Table 2:** Sample demonstrative

	N	%
No loss of radial height	7	10.8%
Loss of radial height up to 2 mm	49	75.4%
Loss of radial height higher than 2 mm	9	13.8%
Total	65	100%

Using the IDEAL classification system for patient evaluation and respecting our inclusion and exclusion criteria, only type-II patients were evaluated. This is because in type I, patients are treated conservatively, whereas in type III, patients require combined methods of osteosynthesis or bone grafting. 17

Two cases of radial height increase were observed, and the parameter returned to the value visualized on the first radiograph in subsequent measurements. Another 2 cases were also observed with only 2 postoperative follow-up radiographs demonstrating an increase in radial height greater than 1 mm (these 4 cases are described with those who did not lose radial height). This phenomenon can be explained by variability in the radiographic technique and has also been observed by other authors in subsequent radiographic follow-ups. 18

## Discussion

Distal radius fractures are among the most common fractures in osteoporotic patients and the older population. With greater fragility, especially in the metaphyseal portion of the bone, fixing these fractures is a challenge. With the advent of anatomical locking plates for the distal radius, which promote angular stability with locking screws, surgeons expect maintenance of reduction and the possibility of early rehabilitation. However, studies 19 show that even with locking plates, there is a loss of postoperative reduction parameters.

Figl et al. 20 in 2009 evaluated 58 patients aged 75 years or older for 13 months, investigating secondary loss of reduction after osteosynthesis with the volar locking plates in patients with distal radius fracture, with no change in volar tilt. Cheng et al. 19 found a mean loss of 1.3 ± 0.9mm in radial height at 1-year follow-up. The present study indicates that 9% of patients had a mean loss in radial height of 1.3 mm, with no change in radial or volar tilt. These data are consistent with the literature on the subject.

Similarly, Cheng et al. 19 evaluated 87 patients with distal radius extra-articular fracture fixed with a locking volar plate, observing a mean radial height loss of 1.3 ± 0.9 mm. According to the author, advanced age, positive ulnar variance, changes in bone densitometry, and a shorter distance between the fracture focus and the radiocarpal joint were associated with loss of radial height. In this study, 89.2% of the patients evaluated had some degree of radial height decrease, with only 13.8% of patients with radial height loss greater than 2 mm.

Our sample had mean age of 54 years and considering that advanced age is a risk factor for the loss of radial height, we observed results consistent with those reported in the literature regarding the mean loss of radial height. One reason for this finding is the severity of the cases we operate on at our institution, as we are a quaternary care center within the health system. Thus, we observed other risk factors acting indirectly, such as bone densitometry, a shorter distance between the fracture focus and the radiocarpal joint, and the degree of fragmentation. 15 21

An article by Thompson 22 described, in its systematic review, the presence of other possible complications, including carpal tunnel syndrome (2–3%), tendon rupture (1–2%), and the need for removal of synthetic material (6%). In addition, a study by Xavier et al. 23 pointed out that loss of radial height was associated with a grip strength deficit and loss of arc motion.

According to the IDEAL classification system, fractures classified as type II exhibit deviation and are considered potentially unstable. They have a high risk of loss of reduction and evolution to vicious consolidation, either due to low bone quality in older patients, high-energy trauma in young people, the presence of joint incongruence, or even associated injuries in any age group. 17 In this context, we can observe that in this group, the loss of radial height during postoperative follow-up had a mean of less than 2 mm.

Farhan et al. 24 demonstrated that, in patients with more complex cases treated with an association of osteosynthesis methods, the mean radial height was 8.5 mm. This represents a loss greater than 2 mm. Therefore, we conclude that in cases defined as type III as per the IDEAL classification, there is a greater chance of radial height.

The present study, having evaluated a good number of patients and used the same locking plate model, gives reliability to the results obtained. In line with the current literature on the subject, corroborating and encouraging the use of a locking plate for distal radius fractures to seek to restore and maintain the anatomical parameters of the region.

However, it is critical to shine a light on the negatives. The loss to follow-up of 42.9% of patients in the initial sample shows that this is difficulty in public services, in addition to the relevant number of patients who were operated on with a non-ideal fracture time (greater than 4 weeks) and were excluded from the sample. In addition, there is a possible selection bias in the profile of fractures and patients operated at this institution (a quaternary service), as well as in the evaluation/subdivision of cases based on their preoperative parameters, which may be analyzed in future articles.

Our article has some positive insights. The first is that a good number of patients were evaluated, which increases the reliability of the results. According to the same locking plate model, interfering as little as possible with the surgical technique and subsequent analysis. Finally, sample homogeneity, as seen in patients classified only as type II as per the IDEAL classification, reduces biases that could confuse the results. However, it is essential to highlight the limitations observed, such as the variation in fracture severity and the short follow-up time. In addition, there was no functional evaluation of the patients correlated with the radiographic parameters, as this was not the main objective of the study and was considered a possibility for a future study. Thus, it is essential to continue monitoring to consolidate results and inform future analyses.

## Conclusion

Although anatomical locking plates of the distal radius offer angular stability to the locking screws, a loss of radial height is expected in the postoperative follow-up. However, in most patients, a loss of less than 2 mm is expected. The present study corroborates the use of this osteosynthesis technique, which is a fundamental tool in the approach of complex fractures of the distal third of the radius, to restore and maintain the anatomy of the region. Finally, more studies are essential for correlational assessment between functionality and radiographic parameters.
